# Supplementary figures and images for: Streptococcal Lancefield polysaccharides are critical cell wall determinants for human Group IIA secreted phospholipase A2 to exert its bactericidal effects
Source: PLoS Pathog. 2018 Oct 15;14(10):e1007348. doi: 10.1371/journal.ppat.1007348 (PMC6201954; doi:10.1371/journal.ppat.1007348)

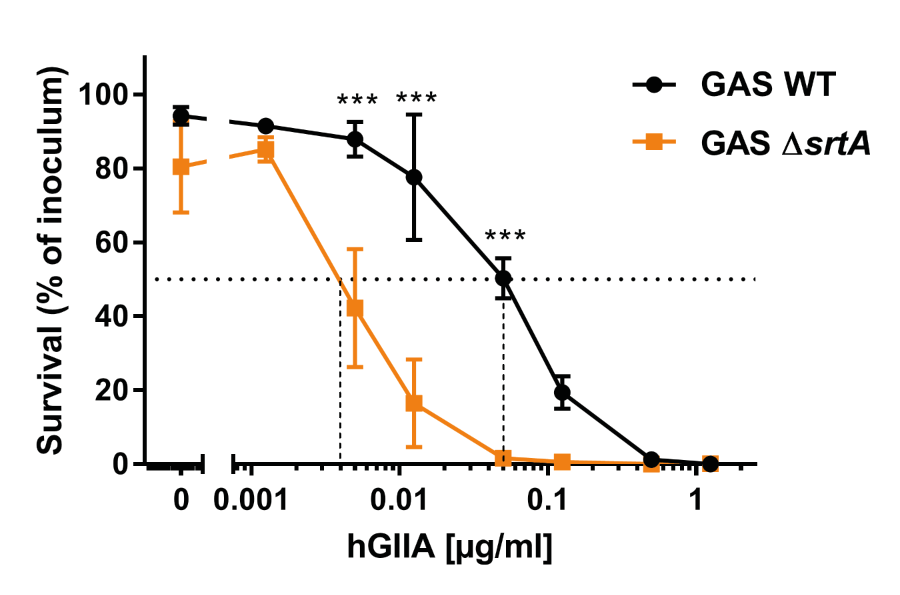

Supplement: S1 Fig — Mutation of srtA renders 5448 more susceptible to hGIIA killing. Data represent mean +/- SD of three independent experiments. ***, p ≤ 0.001. (TIF) [file ppat.1007348.s001.tif]

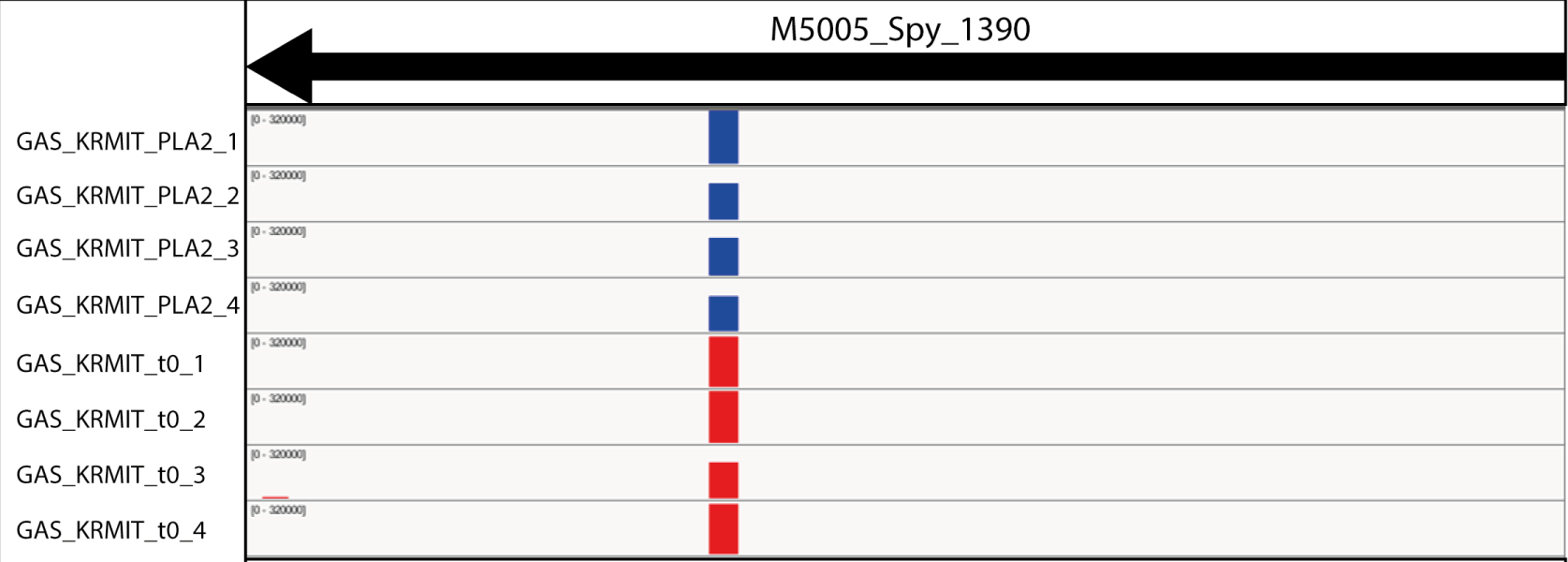

Supplement: S2 Fig — Unusual high number of transposon insertions at one location in the gene M5005_Spy_1390. (TIF) [file ppat.1007348.s002.tif]

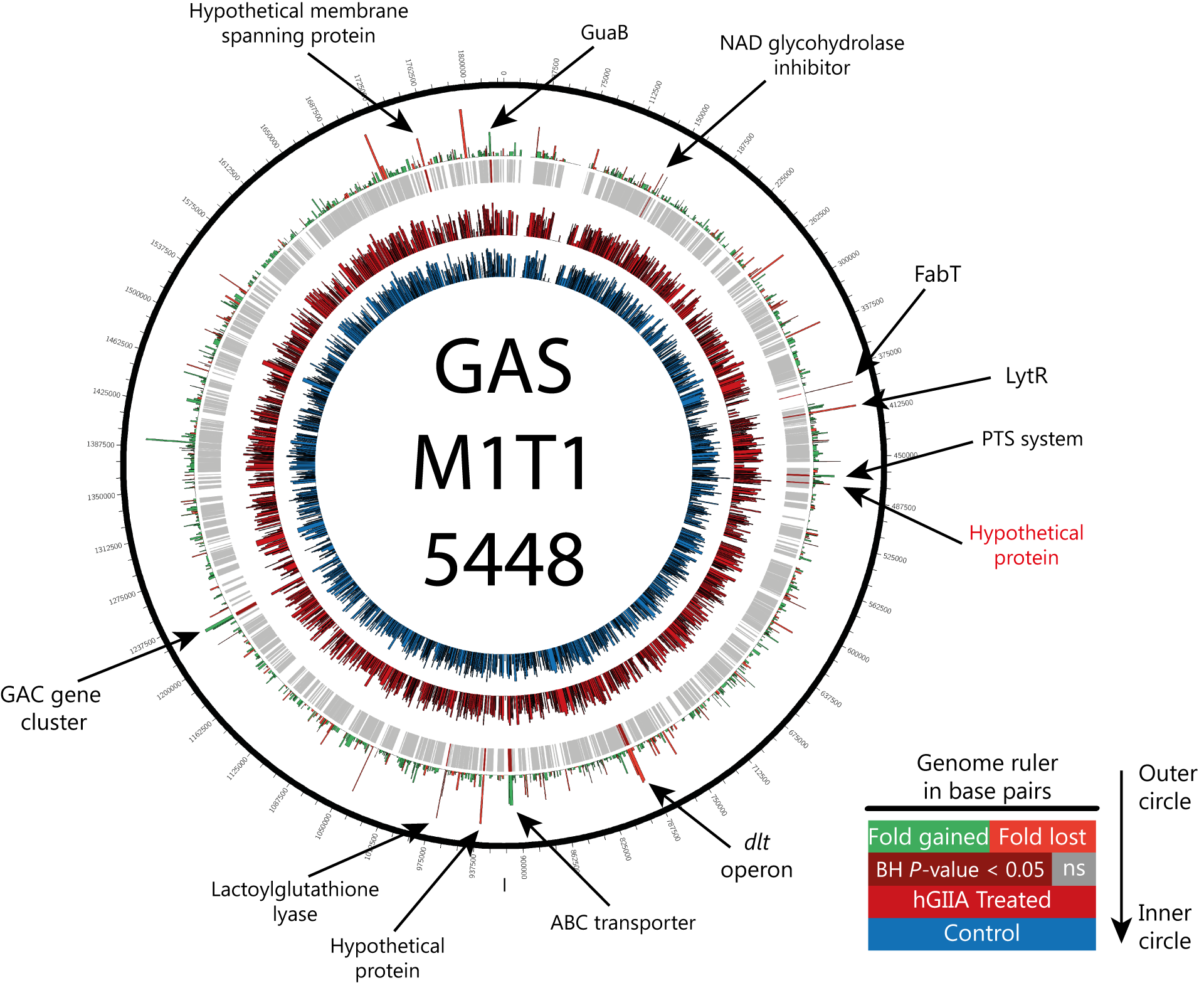

Supplement: S3 Fig — Circos respresentation of the Tn-seq data. Each bar in the inner two circles, where blue is control and red the hGIIA treated, represent the average RKPM value of a gene. The following to circles represent the BH corrected p-value and the fold change in log of the hGIIA treated samples vs control samples. Red bars indicate a fold a respective fold decrease and green bars a respective fold increase of transposon insertions. The gene highlighted in red is M5005_Spy_1390, which showed significant fold change due to unusual high transposon insertions at one specific point in the gene. (TIF) [file ppat.1007348.s003.tif]

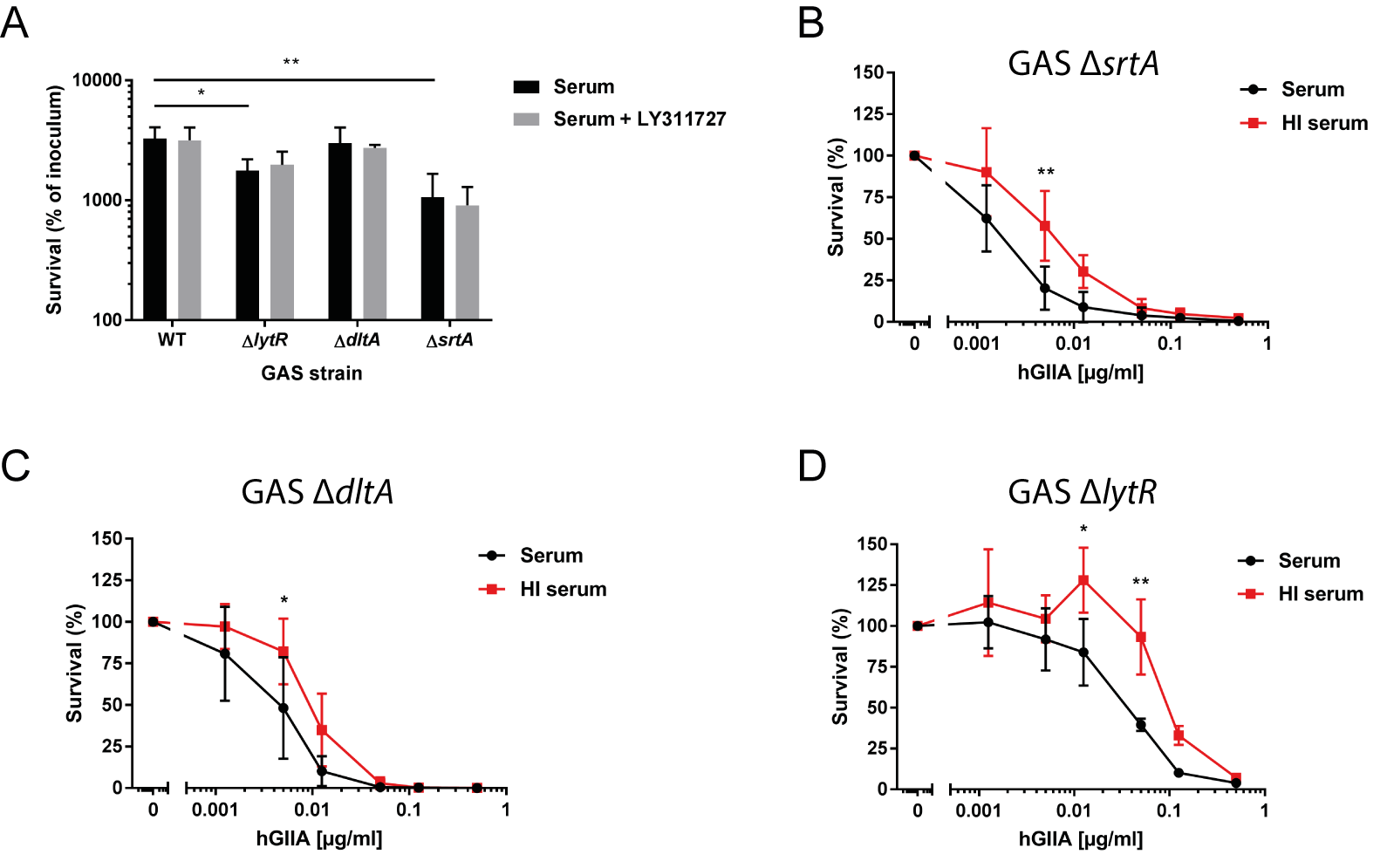

Supplement: S4 Fig — (A) Endogenous hGIIA in serum does not affect growth of GAS. Mutation of lytR and srtA does attenuate GAS growth independent of hGIIA. This heat-labile factor also affects killing of the (B) srtA, (C) dltA, and (D) lytR mutants. Data represent mean +/- SD of three independent experiments, *, p ≤ 0.05; **, p ≤ 0.01. (TIF) [file ppat.1007348.s004.tif]

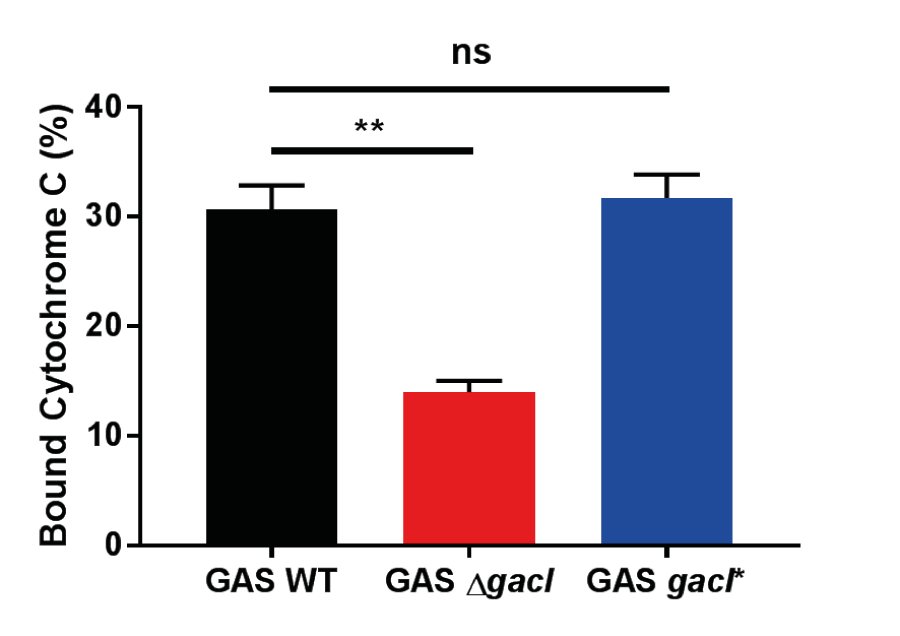

Supplement: S5 Fig — Deletion of gacI affects surface charge of GAS as determined in cationic cytochrome c binding assay. Data represent mean +/- SD of three independent experiments. ns = not significant, **, p ≤ 0.01. (TIF) [file ppat.1007348.s005.tif]

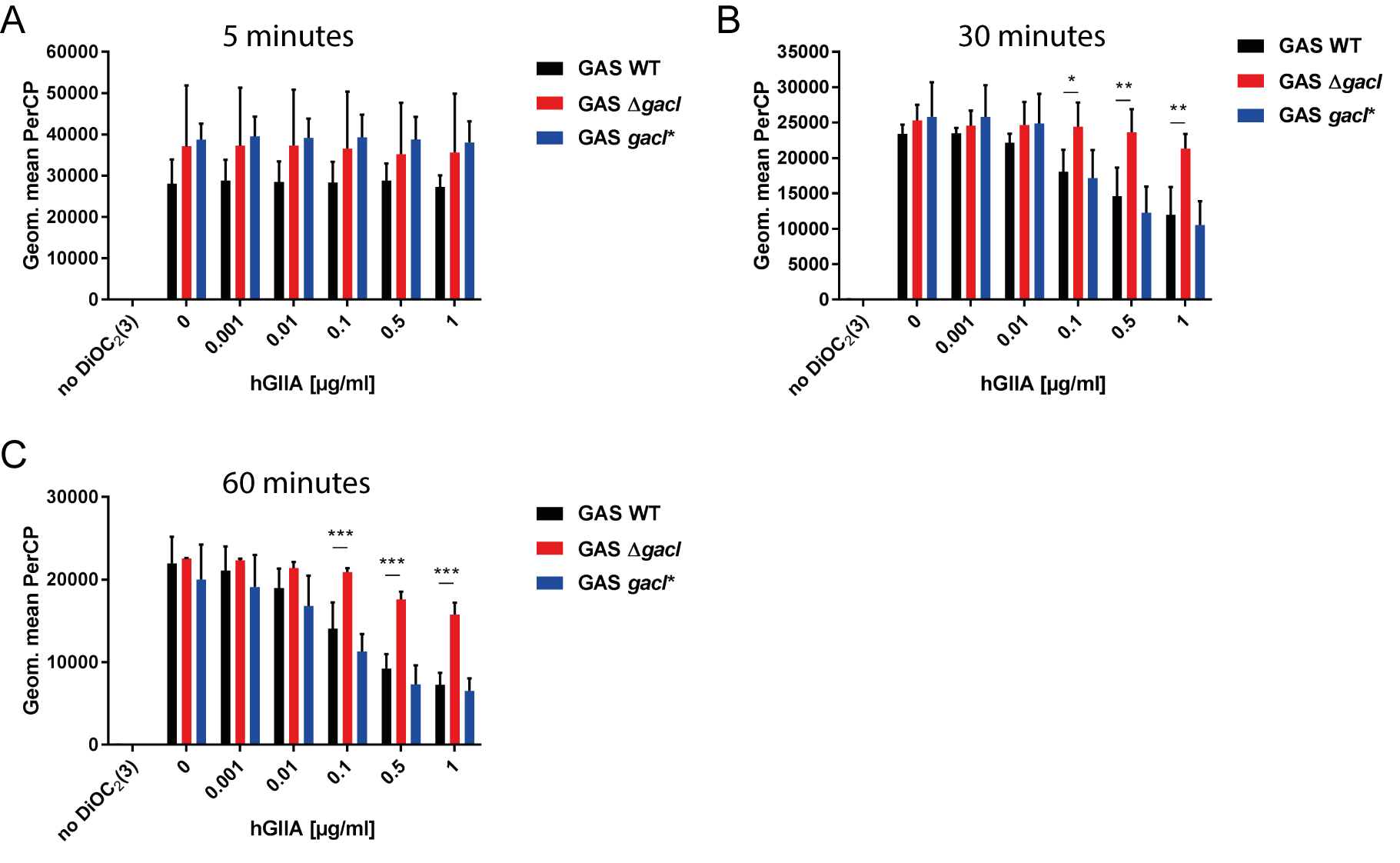

Supplement: S6 Fig — The effect of hGIIA stress on GAS membrane potential after (A) 5 minutes, (B) 30 minutes and (C) 60 minutes. Data represent mean +/- SD of three independent experiments. *, p ≤ 0.05; **, p ≤ 0.01; ***, p ≤ 0.001. (TIF) [file ppat.1007348.s006.tif]

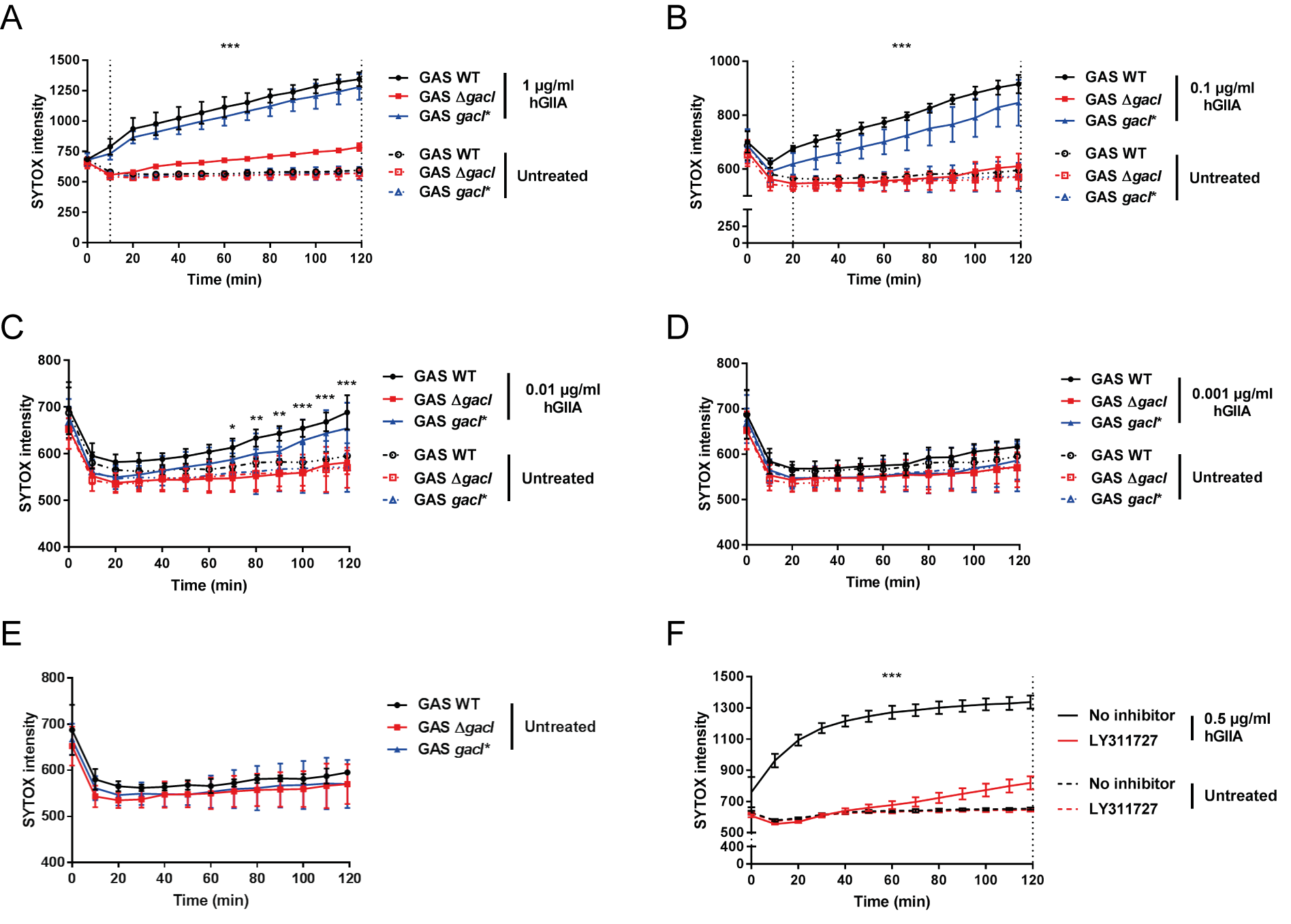

Supplement: S7 Fig — SYTOX influx measured over 120 minutes when GAS strains are incubated with, (A) 1, (B) 0.1, (C) 0.01, (D) 0.001, and (E) 0 μg/ml hGIIA. (F) Addition of 500 μM LY311727 to 0.5 μg/ml hGIIA prevents SYTOX influx. Data represent mean +/- SD of three independent experiments. *, p ≤ 0.05; **, p ≤ 0.01; ***, p ≤ 0.001. (TIF) [file ppat.1007348.s007.tif]

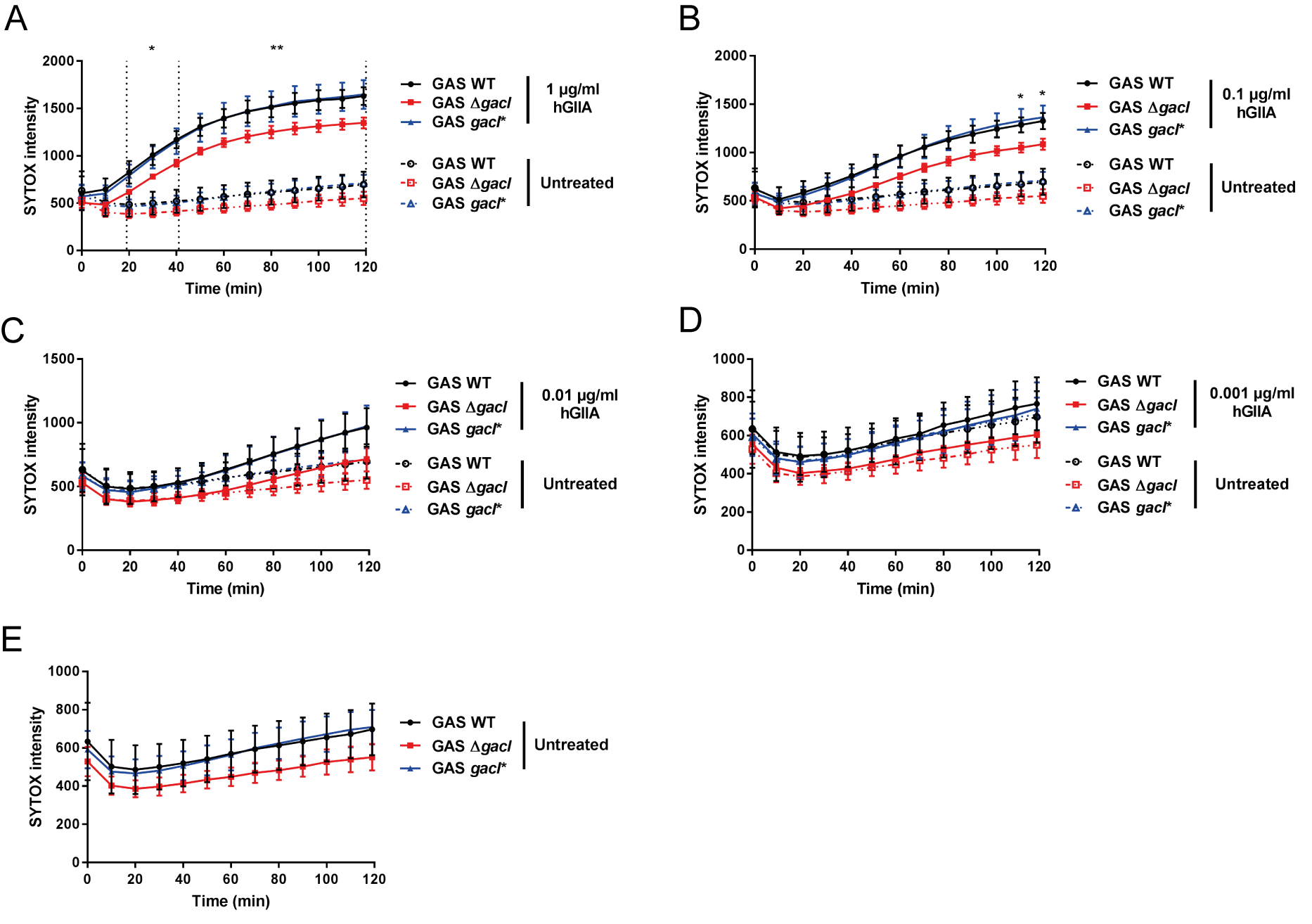

Supplement: S8 Fig — SYTOX influx measured over 120 minutes when protoplast GAS strains are incubated with (A) 1, (B) 0.1, (C) 0.01, (D) 0.001, and (E) 0 μg/ml hGIIA. Data represents mean +/- SD of three independent experiments. *, p ≤ 0.05; **, p ≤ 0.01. (TIF) [file ppat.1007348.s008.tif]

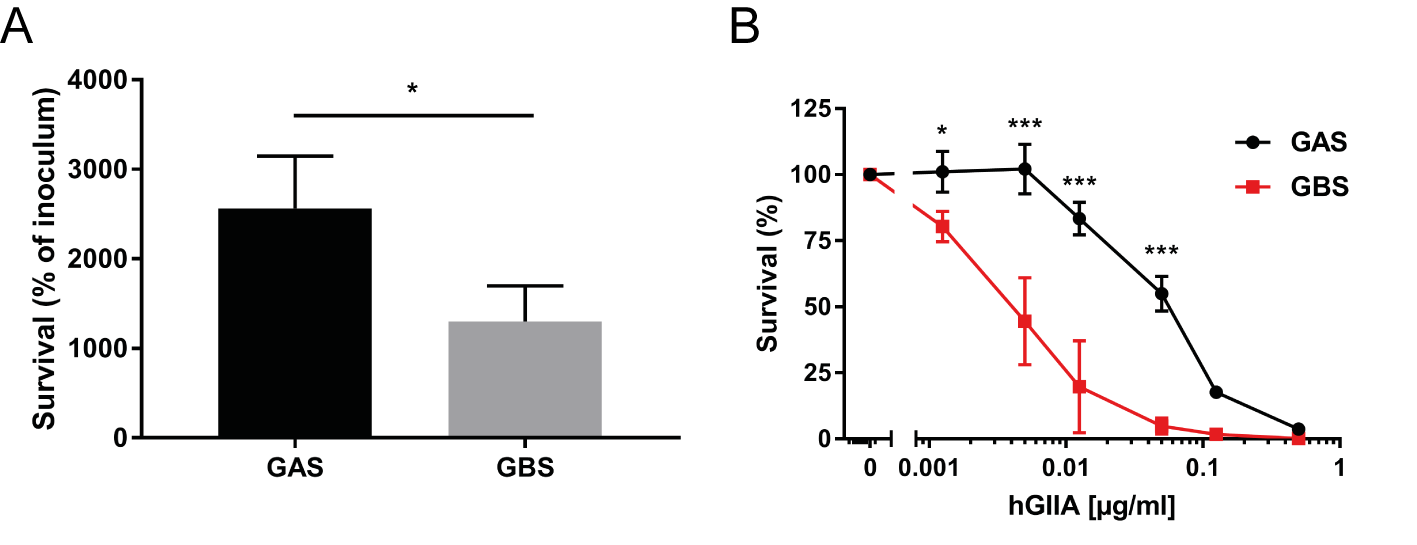

Supplement: S9 Fig — (A) GAS grows faster in human serum compared to GBS. (B) GBS is more susceptible to hGIIA-spiked in serum compared to GAS. Data represent mean +/- SD of three independent experiments. *, p ≤ 0.05; ***, p ≤ 0.001. (TIF) [file ppat.1007348.s009.tif]

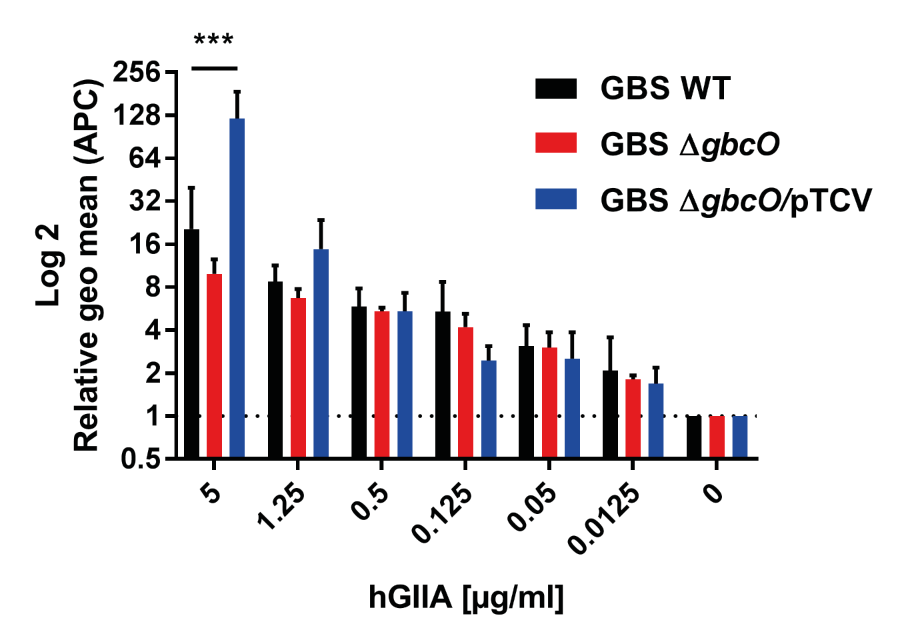

Supplement: S10 Fig — No significant difference in relative hGIIA surface binding of GBS WT and GBS ΔgbcO. Data represent mean +/- SD of three independent experiments. ***, p ≤ 0.001. (TIF) [file ppat.1007348.s010.tif]
